# Using best-worst scaling experiment to understand factors influencing self-medication practices with antimicrobial drugs: A survey of students studying health programs at a tertiary institution in Ghana

**DOI:** 10.1371/journal.pgph.0004748

**Published:** 2025-08-06

**Authors:** Eric Nyarko, Enoch Sakyi-Yeboah, Issah Seidu, Ebenezer Ato Ewusie

**Affiliations:** 1 Department of Statistics and Actuarial Science, School of Physical and Mathematical Sciences, College of Basic and Applied Sciences, University of Ghana, Legon, Accra, Ghana; 2 School of Public Service and Governance, Ghana Institute of Management and Public Administration, Achimota, Accra, Ghana; University of Oxford, UNITED KINGDOM OF GREAT BRITAIN AND NORTHERN IRELAND

## Abstract

Antimicrobial drugs have saved millions of lives, but their widespread use to treat infections has significantly contributed to healthcare challenges, particularly antimicrobial resistance (AMR), which poses a global threat. This study investigated the factors influencing self-medication practices with antimicrobial drugs among health science students at a tertiary institution. A cross-sectional survey was conducted from July to August 2024, using interviewer-administered questionnaires to collect data from 300 students. Participants were selected proportionally based on their disciplines through a random sampling technique. We employed the maximum difference model for data analysis. Our results indicated that 51.67% of participants were male, and 77.93% were between 18 and 25 years old. While 58% of respondents perceived themselves to be in good health, 44.67% reported using antimicrobial drugs without a prescription, with 51.33% having done so in the past year. Ampicillin was the most reported non-prescription antimicrobial; participants typically obtained it from pharmacies (52.33%). The key findings revealed that a good knowledge of antimicrobial drugs was the most significant factor influencing self-medication practices, indicated by a marginal utility estimate (MUE) of 0.6958 and a marginal probability (MP) of 0.1243, with a 95% confidence interval (CI) of 0.6203 to 0.7712. Other important influencing factors included previous knowledge of health conditions (MUE: 0.6205; MP: 0.1153; 95% CI: 0.5448 to 0.6959), previous experiences with the same illness (MUE: 0.4886; MP: 0.1011; 95% CI: 0.4122 to 0.5648), previous use of antimicrobial drugs (MUE: 0.2189; MP: 0.0772; 95% CI: 0.1416 to 0.2959), easy access to over-the-counter antimicrobial drugs (MUE: 0.1711; MP: 0.0736; 95% CI: 0.0938 to 0.2482), and the concept of self-care (MUE: 0.1075; MP: 0.0690; 95% CI: 0.0301 to 0.1848). Conversely, participants tended to trade off frustration with hospital protocols, like long waiting queues/times for medical care (MUE: -0.358; MP: 0.0433; 95% CI: -0.4349 to -0.2815), distance to health facilities (MUE: -0.362; MP: 0.0432; 95% CI: -0.4389 to -0.2855), poor quality of care provided (MUE: -0.374; MP: 0.0427; 95% CI: -0.4506 to -0.2971), and dissatisfaction with healthcare workers’ attitudes (MUE: -0.392; MP: 0.0419; 95% CI: -0.4688 to -0.3155). This study is the first to quantify the factors influencing self-medication practices with antimicrobial drugs among health science students using a best-worst scaling (BWS) statistical design methodology. The findings could inform policy discussions on effective health promotion strategies and regulations for prescribing and dispensing antimicrobials. Such efforts are essential for addressing the issue of AMR in Ghana and other developing countries.

## Introduction

Antimicrobial drugs, including antibiotics, antivirals, antifungals, and antiparasitics [1] have saved millions of lives; however, their widespread use in treating infections has significantly contributed to the increasing healthcare burden and has become a global threat, accounting for approximately 25,000 mortality cases each year [2]. Self-medication refers to the practice of using medication or drugs to treat self-recognized ailments without the guidance of healthcare professionals. According to the World Health Organization (WHO), self-medication encompasses the use of drugs to address self-diagnosed symptoms and the intermittent or continuous use of a prescribed drug for chronic or recurrent diseases [3]. In addition to consultations with pharmacists for advice and medication, examples of self-medication include reusing old prescriptions, utilizing surplus medications, and sharing medications with relatives, family, or friends.

While self-medication can offer benefits such as convenience, cost savings, and reduced resource consumption for minor ailments [4], it also poses risks. Accurate diagnoses are not guaranteed, and the side effects of medications may be unknown. The global increase in self-medication with antimicrobials is recognized as a significant risk factor for AMR, potentially leading to health hazards due to incorrect diagnoses, dosages, formulations, routes of administration, and the risk of adverse drug reactions and drug interactions [5–7]. This issue is particularly prevalent in low- and middle-income countries (LMICs) [8]. A report by WHO [9] revealed that around 80% of antibiotics are used in communities within LMICs, with 20–50% abused for self-medication. Poor oversight and inadequate prescription practices intensify this issue, especially in Ghana, where many healthcare facilities lack the capacity for proper bacterial culture and susceptibility testing [10]. Adverse effects of self-medication include wasteful spending, prolonged suffering, and drug dependence. It can also increase morbidity due to adverse events and heightened resistance among pathogens, posing serious health risks [11,12].

The rise in AMR due to self-medication has garnered significant attention from researchers. Key factors driving self-medication with antibiotics include socioeconomic conditions, lifestyle changes, easy medication access, inefficient healthcare systems, high costs, and uncontrolled drug distribution [13]. Delays in medical care [14], past medication experiences, education level, and age [15] also contribute. Furthermore, individuals’ health knowledge, attitudes, and beliefs influence their self-medication practices [2]. Personal experiences, perceived severity of illness, and family and community norms play a role [16]. Inadequate healthcare facilities, long wait times, negative experiences with providers, and easy access to antibiotics without prescriptions further encourage self-medication [16,17].

Studies on self-medication among university students indicate that about 29% of students in Jordan [18], 26% in India [19], and 55.9% in Saudi Arabia [20] have engaged in self-medication. Additionally, a study involving medical students at a university in Southern Iran found that 42.2% of them practiced self-medication [21]. In Nigeria, 38% of students with a health-related background reported practicing self-medication [22]. In a tertiary institution in Ghana, 56.2% of health-science students engaged in self-medication [2]. In comparison, another study in Ghana noted a prevalence of 55.2% among pharmacy students and 51.1% among non-pharmacy students [23].

Although numerous studies have examined self-medication among university students globally [4,12,20,24–29], relatively few studies focus on this issue in Ghana [14,30]. A scoping review highlighted a gap in the literature regarding the factors influencing self-medication with antibiotics in many LMICs [7]. The review noted that most studies on this topic in LMICs were conducted in Asia, with very few from Africa, particularly Sub-Saharan Africa. Thus, this study aimed to investigate the factors influencing self-medication practices with antimicrobial drugs (such as antibiotics, antivirals, antifungals, and antiparasitics) among health science students at the University of Ghana. To the author’s knowledge, this is the first detailed study employing the best-worst scaling (BWS) methodology, rooted in random utility theory, to examine these factors. Research on self-medication among health students is vital, as this group represents a highly educated and health-conscious segment of society. It is imperative to study self-medication among health science students, as they will be responsible for prescribing medications and working in health education in the future. The findings of this study will be instrumental in informing policy discussions regarding effective health promotion strategies and regulations concerning the prescription and dispensing of antimicrobials, which are essential for addressing the problem of AMR in Ghana and other developing nations.

## Methods

### Ethical approval and consent to participate

Ethical clearance was obtained from the Ethics Committee of the College of Basic and Applied Sciences, University of Ghana (Reference No: ECBAS 063/21–22). Before collecting data, we obtained written consent from all respondents after explaining the purpose of the study. We emphasized that participation in the study was voluntary, and respondents could decline without consequences. We safeguarded respondents’ confidentiality and anonymity using unique identifiers. Participants received airtime as compensation for the time they spent completing the questionnaires.

### Study site

This study was conducted at the University of Ghana, located on the Legon campus (05°39’03“ N 00°11’13” W), approximately 13 kilometers northeast of Accra, the capital city of Ghana. The campus is situated at an altitude ranging from 91 to 122 meters and spans an area of about 13 square kilometers [31]. The University of Ghana features a central administration alongside a collegiate system that includes the International Programs Office, the School of Graduate Studies, and several colleges, such as Basic and Applied Sciences, Education, Health Sciences, and Humanities. With a student population of around 61,000, the university is committed to providing various educational programs, including regular, sandwich, weekend, and distance education options, to cater to the diverse learning needs of its students [32]. This study specifically focused on students from the College of Health Sciences. The College comprises six constituent institutions: the University of Ghana Medical School, the University of Ghana Dental School, the School of Allied Health Sciences, the School of Public Health, the School of Nursing, and the Noguchi Memorial Institute for Medical Research. The first three institutions are located near the Central Administration of the College. They are near the Korle-Bu Teaching Hospital, about 25 kilometers from the main university campus. The remaining three constituent institutions are on this main campus [33].

### Attributes/factors determination

We conducted an extensive literature review to identify an initial list of reasons (so-called factors or attributes) influencing self-medication practices with antimicrobial drugs. We tested the content validity with three scholars with expertise in the subject area to ensure the identified factors aligned with the study’s objectives. We then conducted a focus group discussion with 20 participants to narrow the list to 15 relevant factors for self-medication practices. We grouped the participants to ensure diverse perspectives, including an equal number of males and females, healthcare providers, pharmacists, and health science students offering various health-related programs at various levels or academic years of study. The factors include: Long distance travel to health facilities, frustration with hospital protocols (long waiting queues/times to seek medical care), poor quality of the provided care, good knowledge of antimicrobial drugs, dissatisfaction with hospital workers’ attitudes, poor control of antimicrobial drugs dispensation, easy access to antimicrobial drugs over the counter/pharmacies, previous use of antimicrobial drugs, previous knowledge of health condition, consider minor illness, use of leftover antimicrobial drugs, relatively low cost of purchasing antimicrobial than that of seeking care from a medical doctor, recommendation from a friend/relative, idea of self-care, and previous experience with the same illness.

### Statistical experiment design

This study employed BWS, a cutting-edge method for conducting consumer research across various fields, including healthcare, conflict resolution, environmental sustainability, and general consumer studies [34–39]. We applied a statistical experiment block design to create 15 choice scenarios, each featuring seven options. In these scenarios, every factor appears 7 times across all sets. Notably, each factor is included only once within each choice set generated from the block design. Before conducting the survey, we performed a pilot study to test the questionnaire. This pilot study helped us identify potential challenges and issues that could arise during data collection. We evaluated respondents’ understanding of the different factors and assessed the questionnaire length. Based on the pilot study’s findings, we adjusted the questionnaire to address any issues encountered by respondents to enhance comprehension and reduce cognitive burden and information overload.

### Sample size and data collection

**Fig 1:**
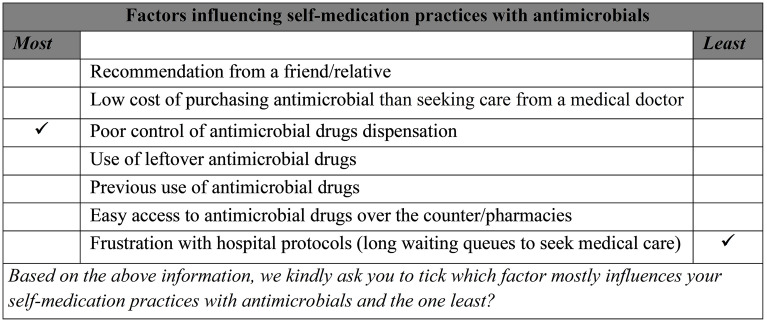
A sample completed BWS scenario presented to respondents.

This cross-sectional study was conducted between July and August 2024 to gather data from 300 students at the University of Ghana enrolled in various health-related programs such as Biomedical and Allied Health Sciences, Medicine and Dentistry, Nursing, Pharmacy, and Public Health. Based on the sample size calculation [40], the minimum required number of respondents was 103, but the anticipated sample size 300 was deemed sufficient for this study. We used the interviewer-questionnaire administration method to collect the data. This approach allowed respondents to seek clarification and share their thoughts, leading to more comprehensive data collection. Respondents were selected based on a proportionate to size from their respective disciplines using a random sampling technique. Enumerators approached respondents in lecture halls before classes began. During data collection, we asked participants to indicate the most and least influential factors affecting their self-medication practices with antimicrobial drugs. Fig 1 displays a sample completed scenario.

Notably, all 300 distributed questionnaires were included in the final analysis, as no data was missing. This was made possible by the presence of enumerators who assisted and guided respondents in completing the survey. We assessed the validity of the responses by examining the consistency of preferences in choice scenarios at the beginning and end of the questionnaire. The inclusion criteria for this study were health science students who had self-administered antimicrobial drugs (i.e., without a prescription). We excluded health science students who refused to consent or had not taken antimicrobial drugs during their health program enrolment from the study.

### Statistical analysis

In this study, we asked participants to identify the most and least influential factors in their self-medication practices with antimicrobial drugs. Their selections from various scenarios, each a significant decision, reveal the trade-offs they are willing to make concerning these factors. We assume that each choice reflects an underlying value or utility for the participants. This utility can be estimated using the maximum difference model [41,42] based on Random Utility Theory, a well-established framework for understanding human decision-making [43]. To formalize this model [37,42], we let φ(\raisebox0.7ex\rule0.1cm0.2pt-5ptλ) and \raisebox0.7ex\rule0.1cm0.2pt-5ptλ indicate the statistical experimental design involving subsets of choice options, and the finite set of theoretically accessible choice options of size three or more in a given best-worst scenario. For any set T∈φ(\raisebox0.7ex\rule0.1cm0.2pt-5ptλ), T⊆\raisebox0.7ex\rule0.1cm0.2pt-5ptλ with |T|≥3, let $Q_{T}\left(a\right)$ represent the probability that the option a is selected as best in set T, $R_{T}\left(b\right)$ the probability that the option b is selected as worst in set T, and $QR_{T}\left(a,b\right)$ the probability of the combination of the alternative’s selection as the best in set T and the alternative’s selection as the worst in set T for any set with b≠a. Here the best choice model assumes that there is a scale γ such that for all b∈T∈φ(\raisebox0.7ex\rule0.1cm0.2pt-5ptλ),


$$Q_{T}\left(b\right)=\frac{e{\mathrm{\backslash nolimits}}^{\gamma \left(b\right)}}{\sum {\mathrm{\backslash nolimits}}_{r\in T}e{\mathrm{\backslash nolimits}}^{\gamma \left(r\right)}},$$ (1)

whereγ(b) is the utility for an option b.

The corresponding model for worst choice assumes that there is a scale υ such that for all a∈T∈φ(\raisebox0.7ex\rule0.1cm0.2pt-5ptλ),


$$R_{T}\left(b\right)=\frac{e{\mathrm{\backslash nolimits}}^{\upsilon \left(b\right)}}{\sum {\mathrm{\backslash nolimits}}_{r\in T}e{\mathrm{\backslash nolimits}}^{\upsilon \left(r\right)}}.$$ (2)

Assuming that all possible pairings of sets of two elements have choice probabilities that satisfy a reasonable requirement for all different pairs, a,b∈T∈φ(\raisebox0.7ex\rule0.1cm0.2pt-5ptλ), and those conditions (1) and (2) are satisfied, then


$$Q_{\left\{a,b\right\}}\left(a\right)=R_{\left\{a,b\right\}}\left(b\right).$$

That is, we have;


$$R_{T}\left(b\right)=\frac{e{\mathrm{\backslash nolimits}}^{-\gamma \left(b\right)}}{\sum {\mathrm{\backslash nolimits}}_{r\in T}e{\mathrm{\backslash nolimits}}^{-\gamma \left(r\right)}}.$$ (3)

When choosing the best option, a choice alternative’s utility is the opposite of its value when choosing the worst option, provided that the choice probabilities for the best (and worst) fulfill (1) and (3). This utility-scale γ is such that for all a,b∈T∈φ(\raisebox0.7ex\rule0.1cm0.2pt-5ptλ), a≠b,


$$QR_{T}\left(a,b\right)=\frac{e{\mathrm{\backslash nolimits}}^{\left[\gamma \left(a\right)-\gamma \left(b\right)\right]}}{\sum {\mathrm{\backslash nolimits}}_{\left\{u,v\right\}\in T}e{\mathrm{\backslash nolimits}}^{\left[\gamma \left(u\right)-\gamma \left(v\right)\right]}}.$$ (4)

In this study, respondents selected the factors from a choice set that most and least influence their self-medication practices with antimicrobial drugs. The frequency of the choices made by participants serves as a metric to compare the relative importance of these factors. We determined statistical significance using a 95% confidence interval (CI). A utility estimate (UE) was considered significant if the 95% CI did not include zero. In cases where CIs were not available, we established statistical significance if the P-value (P) was less than or equal to 0.001, 0.01, or 0.05 [38]. All statistical analyses were conducted using JMP Pro (Version 17.0). A significant positive or negative UE indicates a prioritization or trade-off for a particular factor. It is important to note that the UE reflects the perceived importance of the corresponding levels of each factor. Higher UE values suggest that a specific effect level is considered more important. Standard errors (SE) measure the uncertainty surrounding the parameter estimates for each factor; the better the model fits the data, the smaller the SE will be. The greatest utility difference (GUD) represents the maximum change in utility that can be achieved from a specific factor influencing self-medication practices based on plausible factors identified in the BWS study.

## Results

### Socio-demographic characteristics of the study population

In this study involving 300 health science students, 51.67% identified as male, and most respondents (77.93%) were between 18 and 25 years old. Additionally, 58.0% of the participants believed they were in good health. However, 44.67% had used antimicrobial drugs without a prescription, and 51.33% reported having taken these medications in the past year. Ampicillin was the most commonly used and typically obtained from pharmacies and licensed chemical stores.

### Marginal utility estimates and marginal probability of factors influencing self-medication practices

**Fig 2:**
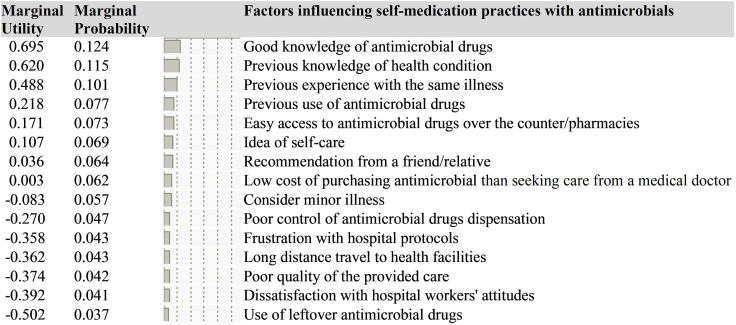
Marginal utility estimates and marginal probability of factors influencing self-medication with antimicrobials.

Fig 2 shows MUE with matching MP values for the factors influencing self-medication with antimicrobials. Larger MUE values imply that the effect level is of greater value. The value attached to the various factors influencing self-medication practices with antimicrobial drugs in order of importance is presented in Fig 2.

### Maximum difference model results

The result of the maximum difference model is displayed in Table 1. The likelihood ratio Chi-square = 1246.73, degree of freedom = 14, and P < 0.00 indicates significant differences in the factors influencing self-medication practices with antimicrobial drugs. All utility estimates significantly influence self-medication practices, except for factors concerning recommendations from friends or relatives and the relatively low cost of purchasing antimicrobial drugs rather than seeking care from a medical doctor. Notably, we treated the use of leftover antimicrobial drugs as the baseline factor.

**Table 1:** Maximum difference model results of factors influencing self-medication practices with antimicrobials.

Attributes	Estimate	SE	Lower 95% CI	Upper 95% CI
Consider minor illness	-0.083	0.039	-0.160	-0.006
Dissatisfaction with hospital workers’ attitudes	-0.392	0.039	-0.468	-0.315
Easy access to antimicrobial drugs over the counter/pharmacies	0.171	0.039	0.093	0.248
Frustration with hospital protocols (long waiting times/queues to seek medical care)	-0.358	0.039	-0.434	-0.281
Good knowledge of antimicrobial drugs	0.695	0.038	0.620	0.771
Idea of self-care	0.107	0.039	0.030	0.184
Long-distance travel to health facilities	-0.362	0.039	-0.438	-0.285
Poor control of antimicrobial drug dispensation	-0.269	0.039	-0.346	-0.192
Poor quality of the provided care	-0.374	0.039	-0.450	-0.297
Previous experience with the same illness	0.488	0.038	0.412	0.564
Previous knowledge of health condition	0.620	0.038	0.544	0.695
Previous use of antimicrobial drugs	0.218	0.039	0.141	0.295
Recommendation from a friend/relative	0.036	0.039	-0.041	0.113
Relatively lower cost of purchasing antimicrobials than that of seeking care from a medical doctor	0.003	0.039	-0.074	0.081
**Model fits**				
L-R Chi-square	1246.73			
AIC	32196.12			
BIC	32285.70			
DF	14			
P-Value	< 0.00			

L-R: likelihood ratio; AICc: Akaike information criteria corrected; BIC: Bayesian information criteria; DF: degree of freedom.

It can be inferred that a good knowledge of antimicrobial drugs has the highest influence on self-medication practices (MUE: 0.695; MP: 0.124; 95% CI: 0.620, 0.771). Other influential factors include previous knowledge of health conditions (MUE: 0.6205; MP: 0.115; 95% CI: 0.544, 0.695), followed by previous experiences with the same illness (MUE: 0.488; MP: 0.101; 95% CI: 0.412, 0.564), previous use of antimicrobial drugs (MUE: 0.218; MP: 0.077; 95% CI: 0.141, 0.295), easy access to these drugs over-the-counter or at pharmacies (MUE: 0.171; MP: 0.073; 95% CI: 0.093, 0.248), and the idea of self-care (MUE: 0.107; MP: 0.069; 95% CI: 0.030, 0.184). However, participants tend to trade off factors such as considering minor illnesses (MUE: -0.083; MP: 0.057; 95% CI: -0.160, -0.006), poor control of antimicrobial drugs dispensation (MUE: -0.270; MP: 0.047; 95% CI: -0.346, -0.192), frustration with hospital protocols (such as long waiting queues/times for medical care) (MUE: -0.358; MP: 0.043; 95% CI: -0.434, -0.281), long-distance travel to health facilities (MUE: -0.362; MP: 0.043; 95% CI: -0.438, -0.285), poor-quality of the provided care (MUE: -0.374; MP: 0.042; 95% CI: -0.450, -0.297), and dissatisfaction with the attitudes of hospital workers (MUE: -0.392; MP: 0.041; 95% CI: -0.468, -0.315).

### Pairwise comparison of factors influencing self-medication practices

The S1 Table (Supplementary material) shows all level comparisons of factors influencing self-medication practices with antimicrobial drugs. Each comparison is the estimated utility difference (DUE) between factors labeling the row and factors labeling the column with their corresponding SE and P-values. Having a good knowledge of antimicrobial drugs has significantly the highest positive utility (DUE: 1.197; P = 2.7e-93) in influencing the practice of self-medication with these drugs compared to all other factors, although it did not differ significantly from previous knowledge of health conditions (P = 0.175). Other significant factors that also showed high utility when presented alongside some factors include previous knowledge of health conditions (DUE: 1.122; P = 6.3e-83), followed by previous experience with the same illness (DUE: 0.990; P = 8e-65) and previous use of antimicrobial drugs (DUE: 0.720; P = 1.2e-35). Easy access to antimicrobial drugs over the counter or from pharmacies (DUE: 0.673; P = 2.1e-31) also demonstrated significant utility, although it did not significantly differ from the idea of self-care (P = 0.270).

The relatively low cost of purchasing antimicrobial drugs compared to seeking medical assistance (P = 0.072) had significant utility (DUE: 0.505; P = 1.8e-18), along with recommendations from friends or relatives (DUE: 0.538; P = 9.6e-21), even though these did not differ significantly from the relatively low cost of purchasing the drugs than that of seeking care from a medical doctor (P = 0.572). Additionally, frustration with hospital protocols, like long waiting queues/times for medical care (DUE: 0.143; P = 0.011), did not significantly differ from the long-distance travel to health facilities (P = 0.944). The quality of care showed a lower but still significant positive utility (DUE: 0.128; P = 0.023).

The results indicate that when previous experience with the same illness is presented alongside previous knowledge of a health condition, study participants tend to trade it off (DUE: -0.131; P = 0.018). Similarly, when participants have easy access to over-the-counter antimicrobial drugs in conjunction with attributes like a good knowledge of these drugs, previous knowledge of the health condition, and previous experience with the same illness, they also tend to trade off (DUE: -0.524; P = 4.6e-20; DUE: -0.449; P = 4.2e-5; and DUE: -0.317; P = 2.76e-8, respectively). Furthermore, participants are inclined to trade off the idea of self-care when it is presented alongside prior knowledge of the health condition (DUE: -0.513; P = 3e-19) and previous experience with the same illness (DUE: -0.381; P = 3.1e-11).

Frustration with hospital protocols, such as long wait times for medical care, is also traded off when presented alongside factors like a good knowledge of antimicrobial drugs (DUE: -1.054; P = 3e-73), the idea of self-care (DUE: -0.465; P = 8.4e-16), previous experience with the same illness (DUE: -0.846; P = 4.3e-48), previous knowledge of the health condition (DUE: -0.978; P = 7.2e-64), previous use of antimicrobial drugs (DUE: -0.577; P = 2.2e-23), recommendations from friends or relatives (DUE: -0.394; P = 7.9e-12), and the relatively low cost of purchasing antimicrobial drugs than that of seeking care from a medical doctor (DUE: -0.361; P = 3.7e-10).

Additionally, long-distance travel to health facilities is traded off when presented alongside previous experience with the same illness (DUE: -0.850; P = 1.4e-48), previous knowledge of the health condition (DUE: -0.982; P = 2.2e-64), previous use of antimicrobial drugs (DUE: -0.581; P = 1.2e-23), recommendations from friends or relatives (DUE: -0.398; P = 5.5e-12), and the relatively low cost of purchasing these drugs than that of seeking care from a medical doctor (DUE: -0.365; P = 2.4e-10). Finally, participants tend to trade off poor quality of the provided care when presented alongside other factors such as previous experience with the same illness (DUE: -0.862; P = 1e-49), previous knowledge of the health condition (DUE: -0.994; P = 1e-65), previous use of antimicrobial drugs (DUE: -0.592; P = 1.6e-24), recommendations from friends or relatives (DUE: -0.410; P = 1.2e-12), and the relatively low cost of purchasing these drugs than that of seeking care from a medical doctor (DUE: -0.273; P = 2.29e-6).

## Discussion

Antimicrobial drugs have saved millions of lives; however, their extensive use in treating infections has significantly added to the healthcare burden, including AMR, which poses a global threat. This cross-sectional study critically investigated the factors influencing self-medication practices with antimicrobial drugs among health science students from various disciplines, such as Biomedical and Allied Health Sciences, Medicine and Dentistry, Nursing, Pharmacy, and Public Health at the University of Ghana.

A key finding of this study was that health science students identified their good knowledge of antimicrobial drugs as the most significant factor influencing their self-medication practices. This was followed by their previous knowledge of health conditions, previous experiences with the same illness, previous use of antimicrobial drugs, easy access to over-the-counter antimicrobial drugs, and the concept of self-care. However, students tended to trade off or were averse to certain factors, such as frustration with hospital protocols, the distance to healthcare facilities, poor quality of the care provided, and dissatisfaction with the attitudes of healthcare workers, as influencing their self-medication practices.

Our findings indicated that health science students cite their good knowledge of antimicrobial drugs as the reason for self-medication. This aligns with findings from a study involving medical students at a university in Southern Iran, which reported that medical knowledge influenced self-medication practices [21]. Similar patterns were observed in studies conducted among healthcare students in Nigeria [25], Jordan [44], Iran [45], and India [46,47], where students felt more confident about practicing self-medication based on their medical/ pharmacological knowledge. However, a contradictory study in Palestine found that medication knowledge was not a significant determinant of self-medication practices with antibiotics among university students [48].

Additionally, previous knowledge of health conditions was noted as a significant reason for engaging in self-medication. This partially aligns with studies in Nepal that report self-medication as common among medical students due to their good educational background and knowledge of diseases [49,50]. Moreover, previous experience with the same illness was also identified in the current study as a reason for seeking self-medication, a finding consistent with research conducted among medical university students in Nepal [50]. In their study, students reported that previous experience with similar illnesses influenced their self-medication. A study has cited familiarity with treatment options as a reason for self-medication among students in Ghana [30]. A meta-analysis and systematic review found that previous experience with similar symptoms was the most reported reason for self-medication among university students in LMICs [51]. Similar patterns have been observed in Pakistan [52], Egypt [53], and Sri Lanka [54], where students used medications to treat similar symptoms based on previous experiences.

Furthermore, reasons why Eritrean students self-medicate with antibiotics included previous successful experiences with these medications [55]. This corroborates our current findings, where previous use of antimicrobial drugs was identified as a reason for engaging in self-medication among health science students. This also aligns with studies in Nepal [49], China [56], Iran [45], and India [57], which found that previous experience with the medication or old prescriptions served as sources of information for self-medication.

The present study found that easy access to antimicrobial drugs over the counter/pharmacies significantly influences self-medication practices. This finding aligns with a previous study conducted among health science students in India, where individuals obtained medications without valid prescriptions [19]. Similarly, a study in Ghana highlighted the easy availability of antibiotics for self-medication [14], with pharmacies identified as the primary source of these drugs [2]. Comparable trends have also been observed in Ethiopia [24], Eritrea [55], China [56], and Qatar [58], where students purchased medications from drug outlets without prescriptions.

In Palestine, a study found that self-care orientation significantly influenced the practice of self-medication with antibiotics among university students [48]. This finding is consistent with the current study, where the idea of self-care also influenced self-medication practices. Indian medical students reported that they viewed self-medication as an integral part of self-care that should be encouraged [47]. In Uganda, students expressed a sense of personal responsibility for their health and well-being through self-medication [59]. A review examining self-medication among physicians and medical students identified self-treatment and self-medication as significant concerns for both groups [60]. The WHO recognizes self-medication as an important aspect of self-care, which can alleviate pressure on healthcare systems and promote more efficient use of medical facilities [61], particularly in LMICs where healthcare services may be scarce [62]. However, it is crucial to adapt WHO guidelines on self-care to local contexts, as self-medication as part of self-care can only be justified with the judicious use of medications [47].

A noteworthy finding from this study was that participants tended to trade off several factors influencing their self-medication practices with antimicrobial drugs. These factors included the consideration of minor illnesses, poor control of antimicrobial drug dispensation, frustrations with hospital protocols, long-distance travel to healthcare facilities, poor quality of care, and dissatisfaction with hospital workers’ attitudes. These findings contrast with several earlier studies. For example, the present study noted that participants tended to trade off frustrations with hospital protocols (such as long queues/times for medical care) as a reason for self-medication. At the same time, previous research in Ghana indicated that long delays and extensive wait times in hospitals and clinics were significant reasons for self-medication among students [14,30]. Again, our results contradict findings in Nepal, where students reported difficulties securing quick appointments [50], and in China, where longer waiting times were common in more developed regions [56] as reasons for self-medication. Other contradictory findings were that the desire to save time or a general lack of time motivated students to self-medicate in Uganda [59], India [46], and Pakistan [52]. A meta-analysis indicated that a lack of available time for seeking medical advice was a common reason for self-medication among students in LMICs due to their heavy academic workloads [51].

Our results revealed that participants traded off the consideration of minor illnesses as a reason for self-medication, which contrasts with studies conducted in Eritrea [55], Egypt [53], Nepal [50], Nigeria [25], Ethiopia [57], Palestine [48], Iran [45], and India [46,47,57], where students commonly cited the minor/mild nature of their ailments as a reason for self-medication. A previous study identified weak enforcement of drug regulations in a previous study conducted in China as influencing self-medication [56]. In contrast, in this study, health science students tended to trade off the poor control of antimicrobial drug dispensation as a reason for self-medication. However, the unregulated sale of medications may continuously trigger self-medication practices [25]. Therefore, it is essential to implement regulations for dispensing antimicrobial drugs [37], as outlined in the Ghana National Action Plan on AMR [63], to combat AMR in the country. Additionally, the Pharmacy Act of 1994 (Act 489) includes guidelines for the dispensing and selling of medications [64], which authorities in the country should enforce. Governments must issue relevant policies and laws prohibiting the sale of medications without a prescription from retail pharmacies [56].

In this study, participants were averse to dissatisfaction with hospital workers’ attitudes as a reason for self-medication. This finding contrasts with research conducted among students at the University of Ghana, where unpleasant attitudes of healthcare providers were noted as a reason for self-medication [30]. Furthermore, distance to health facilities has been highlighted in studies conducted in Nepal [50] and Saudi Arabia [65] as a reason for self-medication among medical students. However, our study found that participants were averse to associating long-distance travel to healthcare facilities with self-medication; this could be due to the proximity of the healthcare facilities at the University of Ghana. Previous research in Uganda reported that the pressure of academic deadlines, such as exams, compels students to seek immediate remedies for health issues. The proximity of drugstores provides a convenient means of obtaining medications quickly, which aligns with their academic demands [59].

Poor quality of care was traded off as a reason for self-medication in this study; in contrast, a previous study noted that busy physician schedules, leading to unavailable appointments, are a reason for self-medication among Bangladeshi undergraduate pharmacy students [66]. In India, medical students engage in self-medication to avoid overcrowding in outpatient departments of hospitals and clinics [47]. In Kuwait, reasons for preferring self-medication over visiting a doctor included a lack of trust in doctors and the limited availability of healthcare services [67]. University health facilities must enhance their service efficiency to build students’ trust and save time, considering their demanding academic schedules [46,51,62].

Although this study is the first to quantify the factors influencing self-medication practices with antimicrobial drugs among health science students using the BWS statistical design methodology, it is important to acknowledge the limitations of this study. The research was uniquely focused on health science students at a specific tertiary institution, without a comparison group from different universities or fields, particularly from non-health sciences. This particular focus, combined with the probability sampling technique employed in this study, may limit the generalizability of the findings to health science students only. An interview-administered survey may introduce interviewer bias, even though participants could self-administer the questionnaire if requested. Furthermore, the study focused on specific factors while excluding other important influences on self-medication practices, such as socioeconomic factors, pharmaceutical advertising in the media, inadequate respect for patient privacy, and access to medical staff. These exclusions could skew the utility estimates. Therefore, future research should incorporate these factors to mitigate biases related to the excluded factors.

In conclusion, this study provides essential insights to guide policy discussions on effective health promotion strategies and regulations regarding prescribing antimicrobials in Ghana and other developing countries. The findings emphasize the urgent need for a national commitment to address the overuse, misuse, and resistance to antimicrobial drugs. This commitment requires extensive health education to encourage behavioral changes and to enforce strict measures against the irrational use of these medications. Comprehensive awareness campaigns are vital for educating health science students about the risks associated with self-medication and the appropriate use of antimicrobials. It is also important to enforce laws regarding medication dispensing to regulate access to antimicrobial drugs, thereby reducing instances of self-medication with these substances. These measures are crucial for promoting responsible and informed use of antimicrobials and will contribute to global efforts to combat AMR. As future healthcare professionals, health science students have a key role in this critical battle.

## Supporting information

S1 Table All levels comparison of factors influencing self-medication practices with antimicrobials. (DOCX)
