# Supplementary material for: Using best-worst scaling experiment to understand factors influencing self-medication practices with antimicrobial drugs: A survey of students studying health programs at a tertiary institution in Ghana
Source: PLOS Glob Public Health. 2025 Aug 6;5(8):e0004748. doi: 10.1371/journal.pgph.0004748 (PMC12327658; doi:10.1371/journal.pgph.0004748)
Supplement: S1 Table — (DOCX) [file pgph.0004748.s001.docx]

**Supporting information:**

S1 Table: All levels comparison of factors influencing self-medication practices with antimicrobials

| Difference (Row-Column)  Standard Error of Difference  Wald p-Value | Consider minor illness | Dissatisfaction with hospital workers' attitudes | Easy access to antimicrobial drugs over the counter/pharmacies | Frustration with hospital protocols (long waiting queues/times to seek medical care) | Good knowledge of antimicrobial drugs | Idea of self-care | Long distance travel to health facilities | Poor control of antimicrobial drugs dispensation | Poor quality of the provided care | Previous experience with the same illness | Previous knowledge of health condition | Previous use of antimicrobial drugs | Recommendation from a friend/relative | Relatively low cost of purchasing antimicrobial than that of seeking care from a medical doctor | Use of leftover antimicrobial drugs |
| --- | --- | --- | --- | --- | --- | --- | --- | --- | --- | --- | --- | --- | --- | --- | --- |
| Consider minor illness | 0  0  . | 0.308  0.057  7.89e-8 | -0.254  0.057  0.000 | 0.274  0.057  1.82e-6 | -0.779  0.057  1.4e-41 | -0.191  0.057  0.000 | 0.278  0.057  1.29e-6 | 0.186  0.057  0.001 | 0.290  0.057  4.68e-7 | -0.572  0.057  3.7e-23 | -0.704  0.056  1.7e-34 | -0.302  0.057  1.69e-7 | -0.119  0.057  0.038 | -0.087  0.057  0.133 | 0.418  0.057  3.2e-13 |
| Dissatisfaction with hospital workers' attitudes | -0.308  0.057  7.89e-8 | 0  0  . | -0.563  0.057  2.2e-22 | -0.033  0.056  0.551 | -1.088  0.057  1e-77 | -0.499  0.057  6.8e-18 | -0.03  0.057  0.599 | -0.122  0.057  0.031 | -0.018  0.056  0.748 | -0.880  0.057  9.6e-52 | -1.012  0.057  7.2e-68 | -0.611  0.057  6.1e-26 | -0.428  0.057  1.3e-13 | -0.395  0.057  7.2e-12 | 0.109  0.056  0.052 |
| Easy access to antimicrobial drugs over the counter/pharmacies | 0.254  0.057  0.000 | 0.563  0.057  2.2e-22 | 0  0  . | 0.529  0.057  5.4e-20 | -0.524  0.056  4.6e-20 | 0.063  0.057  0.270 | 0.533  0.057  2.9e-20 | 0.440  0.057  2.6e-14 | 0.545  0.057  4.6e-21 | -0.317  0.057  2.76e-8 | -0.449  0.057  4.2e-15 | -0.047  0.057  0.405 | 0.134  0.057  0.019 | 0.167  0.057  0.003 | 0.673  0.057  2.1e-31 |
| Frustration with hospital protocols (long waiting queues/times to seek medical care) | -0.274  0.057  1.82e-6 | 0.033  0.056  0.551 | -0.529  0.057  5.4e-20 | 0  0  . | -1.054  0.057  3e-73 | -0.465  0.057  8.4e-16 | 0.003  0.057  0.944 | -0.088  0.057  0.121 | 0.015  0.056  0.782 | -0.846  0.057  4.3e-48 | -0.978  0.057  7.2e-64 | -0.577  0.057  2.2e-23 | -0.394  0.057  7.9e-12 | -0.361  0.057  3.7e-10 | 0.143  0.056  0.011 |
| Good knowledge of antimicrobial drugs | 0.779  0.057  1.4e-41 | 1.088  0.057  1e-77 | 0.524  0.056  4.6e-20 | 1.054  0.057  3e-73 | 0  0  . | 0.588  0.056  7.8e-25 | 1.058  0.057  8.8e-74 | 0.965  0.057  3.6e-62 | 1.069  0.057  2.9e-75 | 0.207  0.055  0.000 | 0.075  0.055  0.175 | 0.476  0.056  4.3e-17 | 0.659  0.056  1.2e-30 | 0.692  0.057  2.5e-33 | 1.197  0.057  2.7e-93 |
| Idea of self-care | 0.190  0.057  0.000 | 0.499  0.057  6.8e-18 | -0.063  0.057  0.270 | 0.465  0.057  8.4e-16 | -0.588  0.056  7.8e-25 | 0  0  . | 0.469  0.057  4.9e-16 | 0.377  0.057  7.2e-11 | 0.481  0.057  8.7e-17 | -0.381  0.057  3.1e-11 | -0.513  0.056  3e-19 | -0.111  0.057  0.053 | 0.071  0.057  0.218 | 0.103  0.057  0.072 | 0.609  0.057  6.2e-26 |
| Long distance travel to health facilities | -0.278  0.057  1.29e-6 | 0.029  0.057  0.599 | -0.533  0.057  2.9e-20 | -0.004  0.057  0.944 | -1.058  0.057  8.8e-74 | -0.469  0.057  4.9e-16 | 0  0  . | -0.092  0.057  0.105 | 0.011  0.057  0.837 | -0.850  0.057  1.4e-48 | -0.982  0.057  2.2e-64 | -0.581  0.057  1.2e-23 | -0.398  0.057  5.5e-12 | -0.365  0.057  2.4e-10 | 0.139  0.056  0.013 |
| Poor control of antimicrobial drugs dispensation | -0.186  0.057  0.001 | 0.122  0.057  0.031 | -0.440  0.057  2.6e-14 | 0.088  0.057  0.121 | -0.965  0.057  3.6e-62 | -0.377  0.057  7.2e-11 | 0.092  0.057  0.105 | 0  0  . | 0.104  0.057  0.068 | -0.758  0.057  4.7e-39 | -0.890  0.057  2e-53 | -0.488  0.057  3.1e-17 | -0.305  0.057  1.16e-7 | -0.273  0.057  2.29e-6 | 0.232  0.057  4.59e-5 |
| Poor quality of the provided care | -0.290  0.057  4.68e-7 | 0.018  0.056  0.748 | -0.545  0.057  4.6e-21 | -0.015  0.056  0.782 | -1.069  0.057  2.9e-75 | -0.481  0.057  8.7e-17 | -0.011  0.057  0.837 | -0.104  0.057  0.068 | 0  0  . | -0.862  0.057  1e-49 | -0.994  0.057  1e-65 | -0.592  0.057  1.6e-24 | -0.410  0.057  1.2e-12 | -0.377  0.057  6.5e-11 | 0.128  0.056  0.023 |
| Previous experience with the same illness | 0.572  0.057  3.7e-23 | 0.880  0.057  9.6e-52 | 0.317  0.057  2.76e-8 | 0.846  0.057  4.3e-48 | -0.207  0.055  0.000 | 0.381  0.057  3.1e-11 | 0.850  0.057  1.4e-48 | 0.758  0.057  4.7e-39 | 0.862  0.057  1e-49 | 0  0  . | -0.131  0.056  0.018 | 0.269  0.057  2.55e-6 | 0.452  0.057  3.6e-15 | 0.485  0.057  4.1e-17 | 0.990  0.057  8e-65 |
| Previous knowledge of health condition | 0.703  0.056  1.7e-34 | 1.012  0.057  7.2e-68 | 0.449  0.057  4.2e-15 | 0.978  0.057  7.2e-64 | -0.075  0.055  0.17508 | 0.512  0.056  3e-19 | 0.982  0.057  2.2e-64 | 0.890  0.057  2e-53 | 0.994  0.057  1e-65 | 0.131  0.056  0.018 | 0  0  . | 0.401  0.056  1.9e-12 | 0.584  0.057  2.2e-24 | 0.616  0.057  6.8e-27 | 1.122  0.056  6.3e-83 |
| Previous use of antimicrobial drugs | 0.302  0.057  1.69e-7 | 0.611  0.057  6.1e-26 | 0.047  0.057  0.405 | 0.577  0.057  2.2e-23 | -0.477  0.056  4.3e-17 | 0.111  0.057  0.053 | 0.581  0.057  1.2e-23 | 0.488  0.057  3.1e-17 | 0.592  0.057  1.6e-24 | -0.269  0.057  2.55e-6 | -0.401  0.056  1.9e-12 | 0  0  . | 0.182  0.057  0.001 | 0.215  0.057  0.000 | 0.720  0.057  1.2e-35 |
| Recommendation from a friend/relative | 0.119  0.057  0.038 | 0.428  0.057  1.3e-13 | -0.134  0.057  0.019 | 0.394  0.057  7.9e-12 | -0.659  0.056  1.2e-30 | -0.071  0.057  0.218 | 0.398  0.057  5.5e-12 | 0.305  0.057  1.16e-7 | 0.410  0.057  1.2e-12 | -0.452  0.057  3.6e-15 | -0.584  0.057  2.2e-24 | -0.182  0.057  0.001 | 0  0  . | 0.032  0.057  0.572 | 0.538  0.057  9.6e-21 |
| Relatively low cost of purchasing antimicrobial than that of seeking care from a medical doctor | 0.087  0.057  0.133 | 0.395  0.057  7.2e-12 | -0.167  0.057  0.003 | 0.361  0.057  3.7e-10 | -0.692  0.057  2.5e-33 | -0.104  0.057  0.072 | 0.365  0.057  2.4e-10 | 0.273  0.057  2.29e-6 | 0.377  0.057  6.5e-11 | -0.485  0.057  4.1e-17 | -0.617  0.057  6.8e-27 | -0.215  0.057  0.000 | -0.032  0.057  0.572 | 0  0  . | 0.505  0.057  1.8e-18 |
| Use of leftover antimicrobial drugs | -0.418  0.057  3.2e-13 | -0.109  0.056  0.052 | -0.673  0.057  2.1e-31 | -0.143  0.056  0.011 | -1.198  0.057  2.7e-93 | -0.609  0.057  6.2e-26 | -0.139  0.056  0.013 | -0.232  0.057  4.59e-5 | -0.128  0.056  0.023 | -0.990  0.057  8e-65 | -1.122  0.056  6.3e-83 | -0.721  0.057  1.2e-35 | -0.538  0.057  9.6e-21 | -0.505  0.057  1.8e-18 | 0  0  . |
